# Beneficial effects of whole-body cryotherapy on glucose homeostasis and amino acid profile are associated with a reduced myostatin serum concentration

**DOI:** 10.1038/s41598-021-86430-9

**Published:** 2021-03-29

**Authors:** Marta Kozłowska, Jakub Kortas, Małgorzata Żychowska, Jędrzej Antosiewicz, Klaudia Żuczek, Silvia Perego, Giovanni Lombardi, Ewa Ziemann

**Affiliations:** 1 Department of Physiology and Biochemistry, Gdańsk University of Physical Education and Sport, Gdańsk, Poland; 2 Department of Sport, Gdansk University of Physical Education and Sport, Gdańsk, Poland; 3 Department of Sport, Bydgoszcz Kazimierz Wielki University, Bydgoszcz, Poland; 4 Department of Bioenergetics and Physiology of Exercise, Medical University of Gdansk, Gdańsk, Poland; 5 Department of Medical Biology and Genetics, Faculty of Biology, University of Gdańsk, Gdańsk, Poland; 6 Laboratory of Experimental Biochemistry and Molecular Biology, IRCCS Istituto Ortopedico Galeazzi, Milano, Italy; 7 Department of Athletics, Strength and Conditioning, Poznan University of Physical Education, Poznan, Poland

**Keywords:** Physiology, Health care

## Abstract

The study investigated the effect of single and chronic (10 sessions) whole-body cryotherapy (WBC; 3-min, − 110 °C) on amino acid (AA) profile, myostatin, fibroblast growth factor 21 (FGF21), and concentrations of brain-derived neurotrophic factor (BDNF), irisin and adiponectin in relation to glucose homeostasis. Thirty-five, healthy men were randomly split into experimental (young: 28 ± 7 years and middle-aged: 51 ± 3 years) and control groups. Blood samples were taken before and 1 h after the first and last (10th) WBC session. Baseline myostatin correlated significantly with visceral fat area, glucose, insulin, HOMA-IR and irisin (all *p* < 0.05). The single session of WBC induced temporary changes in AA profile, whereas chronic exposure lowered valine and asparagine concentrations (*p* < 0.01 and *p* = 0.01, respectively) compared to the baseline. The chronic WBC reduced fasting glucose (*p* = 0.04), FGF21 (− 35.8%, *p* = 0.06) and myostatin (-18.2%, *p* = 0.06). Still, the effects were age-dependent. The decrease of myostatin was more pronounced in middle-aged participants (*p* < 0.01). Concentrations of irisin and adiponectin increased in response to chronic WBC, while BDNF level remained unchanged. By improving the adipo-myokine profile, chronic WBC may reduce effectively the risk of the metabolic syndrome associated with hyperinsulinemia, increased levels of valine and asparagine, and muscle atrophy.

## Introduction

Insulin resistance (IR) occurs when higher circulating insulin levels are necessary to achieve the integrated glucose-lowering response^1^. IR results in a compensatory increased release of insulin by pancreatic β-cells and hyperinsulinemia, which is thought to precede the development of type 2 diabetes (T2DM) by 10 to 15 years^2^. Obesity, age and physical inactivity are the most prominent factors exacerbating the risk of developing IR^3^. These factors are codependent. Ageing is associated with a reduced activity, which contributes to lower total energy expenditure^4^ and may lead to fat tissue accumulation^5^, especially visceral fat area (VFA). This condition significantly affects development of the age-related IR^6^. Routine screening tests including fasting glucose concentration and glycated hemoglobin (HbA1C) are most commonly used to detect this condition^7^. The oral glucose tolerance test may also be applied for this purpose, but is performed less frequently due to being poorly tolerated by the patients as well as being time consuming^8^.

Serum amino acids (AA’s) are considered to be useful laboratory biomarkers in detecting early disruptions of glucose homeostasis^9^. Serving as an energy source, AA’s can be used for gluconeogenesis during catabolic states^10^, and influence insulin and glucagon secretion^11^. Increased levels of AA’s have been observed in all stages of diabetes, including early pre-diabetic IR^12^. Insulin reduces concetrations of amino acids in circulation by stimulating their transport to cells^13^. In particular, the elevated circulating branched-chain amino acids (BCAA’s) are considered to be reliable predictors of the T2DM development in normoglycemic subjects^14^. A cross-sectional study including both normoglycemic and T2DM individuals demonstrated higher concentrations of serum BCAA’s and also the aromatic AA’s (tyrosine and phenylalanine) in individuals with impaired fasting glycaemia and IR^15^. The authors reported a decrease in glycine in all T2DM individuals, contrary to the observed increase of AA’s after the meal^15^. This result was attributed to an increased hepatic clearance of postprandial glycine to replenish a conjugated bile acid pool in the gall bladder^16^. Likewise, increased plasma AA’s levels of alanine, proline and glutamate/glutamine were observed in a group of 263 men with different stages of diabetes, including early prediabetic IR^12^.

Due to changes in AA’s concentrations and inhibited insulin action, individuals with IR may also exhibit skeletal mass disfunction and obesity related sarcopenia^17^. Myostatin is one of the factors which contributes to the development of sarcopenia^18^. It is a skeletal muscle-derived member of the transforming growth factor β superfamily, which inhibits protein synthesis via an impaired mammalian target of rapamicyn (mTOR) signaling^19^. Circulating myostatin was previously demonstrated to be correlated with indices of IR^20^. A study in animal models showed that blocking the myostatin receptor induced an elevation of brown adipose tissue (BAT), an improvement of its mitochondrial function, and better cold tolerance, which altogether contributed to an enhanced energy expenditure^21^. Similarly, myostatin propeptide which inhibits its activity prevents the development of diet-induced obesity and insulin resistance in transgenic animals^17^.

Together with physical activity^22^, cold exposure might improve insulin sensitivity and counteract the inflammatory status associated with obesity. By increasing peripheral insulin sensitivity as well as BAT mass and activity, cold-induced adaptive thermogenesis may be a potential therapy for T2DM^23^. Similar to cold water immersion^24^, WBC reduces superficial body temperature leading to changes in tissue blood flow. It does so by means of vasoconstriction at the skin and an increased metabolic rate caused by shivering to maintain a constant core temperature (around 37 °C)^25,26^, ultimately affecting the expression of myokines^27^ and adipokines^28^. These physiological responses provide a theoretical base for applying cold exposure as a possible therapeutic strategy in individuals with metabolic diseases^29^.

Health benefits of cold exposure are releated to shifts in fibroblast growth factor 21 (FGF21) and irisin^30,31^. It has been proven that the secretion of FGF21 is stimulated by nonshivering thermogenesis and irisin, in turn, by shivering thermogenesis^30^. FGF21 regulates expression of genes involved in gluconeogenesis, lipogenesis, lipolysis and fatty acid oxidation^32^. It is also a metabolic regulator with anti-diabetic properties capable of stimulating enhanced glucose uptake in adipocytes^33^. FGF21 enhances energy expenditure by increasing the core body temperature and decreasing the respiratory quotient^34^. Dulian et al. (2015) noted an increase of irisin level in response to 10 sessions of WBC in obese, inactive men, which was also positively correlated withsubcutaneous fat tissue^31^.

Data on the influence of cold exposure on AA profile’s and myostatin are limited^35^. We previously reported that the effect of WBC on adipokines depended on participants’ cardiorespiratory fitness^36^ , expressed in relative maximal oxygen uptake (VO_2_max). Therefore, for this study, we recruited only men with comparable levels of aerobic capacity. As such, the main purpose was to examine whether both acute and chronic WBC affected changes in blood AA’s and myostatin concentration and the secondary purpose was to establish whether the induced changes were be associated with glucose homeostasis. We hypothesized that both a single and chronic WBC exposures would induce an imporvement in glucose metabolism, related to changes in blood myokines and adipokines concentrations, which would allow considering WBC as a preventative strategy against IR and development of T2DM.

## Results

**Figure 1:**
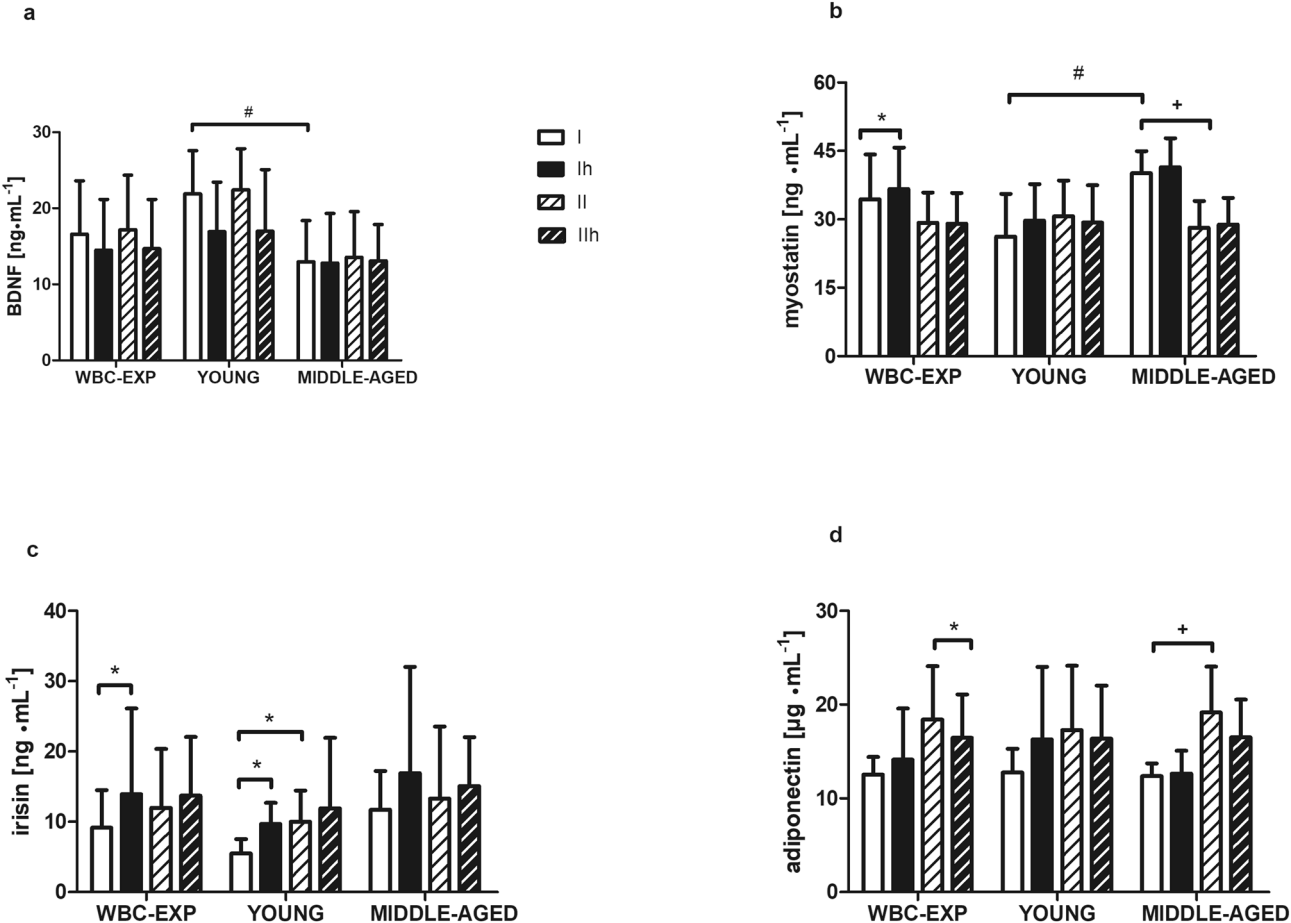
Group- and age-related changes post a single session of the WBC in concentrations of (**a**) BDNF; (**b**) myostatin; (**c**) irisin and (**d**) adiponectin; recorded before (I) and 1 h after the first (Ih) as well as before (II) and 1 h after the last (IIh) WBC session. WBC-EXP (n = 22) included young (YG, n = 9) and middle aged (MG, n = 13) participants. Data are presented as mean ± SD; *statistical significance in the group; #statistical difference between groups at a time point, ^**+**^statistical significance in the group MG vs WBC-CON.

**Figure 2:**
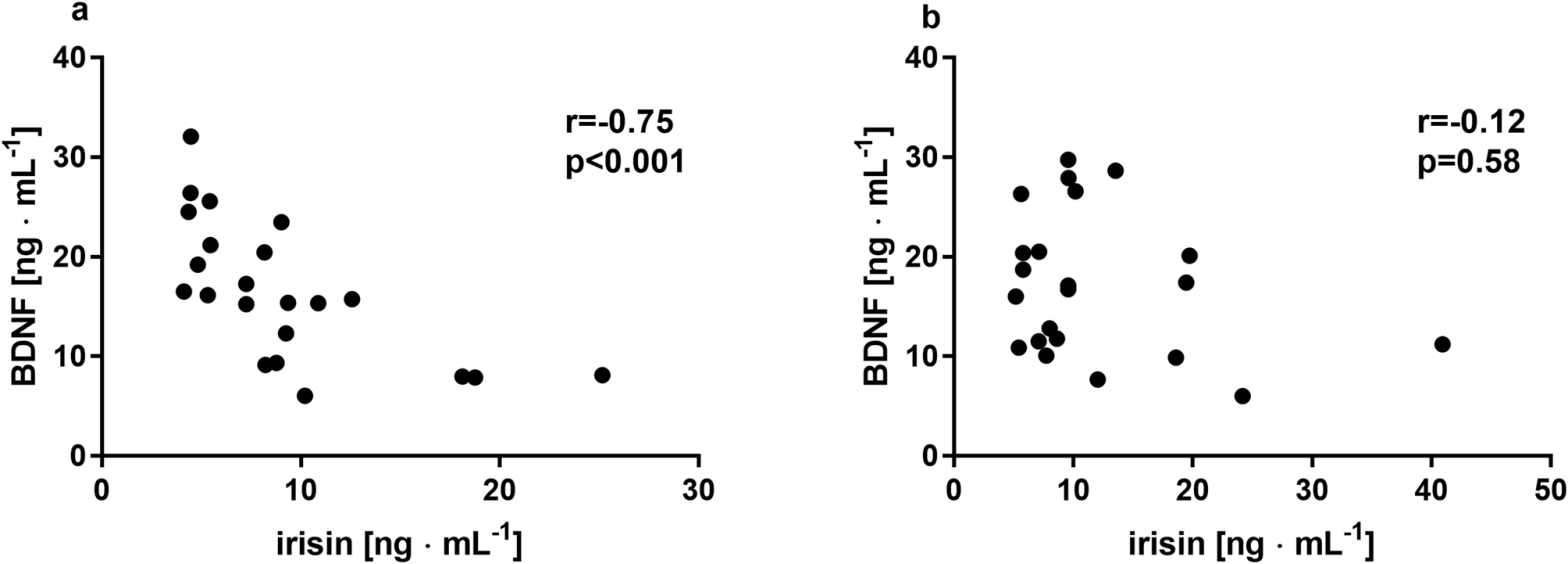
Correlation coefficients between BDNF and irisin in the WBC-EXP (n = 22) group (**a**) prior to and (**b**) after chronic WBC. Values are Spearman correlations, significant at *p* < 0.05.

Significant differences in measured insulin sensivity indicators were noted among participants at baseline. Lower glucose (95.7 ± 8.6 vs 106.5 ± 7.0 mg∙mL^-1^; *p* = 0.002), insulin concentrations (6.3 ± 2.5 vs 9.0 ± 2.1 µU∙mL^-1^; *p* = 0.01) and HOMA-IR (0.8 vs 1.2) were evident in younger participants (YG) compared to middle-aged individuals respectively (MG; supplementary Table S1). These differences were also visible in myokine concentrations. At baseline, BDNF was significantly higher in YG than in MG subjects (*p* = 0.01), while the trend was opposite for myostatin (*p* < 0.01; Fig. 1a,b). Conversely, irisin and adiponectin concentrations did not differ at baseline between the two groups (Fig. 1c,d). In WBC-EXP group, baseline concentrations of BDNF and irisin was negatively correlated (r = -0.75, *p* < 0.01; Fig. 2a), which was not observed at the end of chronic WBC (r = -0.12; *p* = 0.58; Fig. 2b). In turn, irisin concentration correlated positively at baseline with the amount of fat tissue (percentage of body fat, PBF% as well in absolute kilograms) only in MG subjects (r = 0.58, *p* = 0.01; supplementary Table S2).

In the whole group of participants myostatin concentration, regardless of the age, correlated significantly with VFA (r = 0.70, *p* < 0.01), glucose homeostasis indicators such as glucose (r = 0.69, *p* = 0.00), insulin (r = 0.46, *p* = 0.01) and HOMA-IR (r = 0.53, *p* < 0.01) and irisin (r = 0.65, *p* < 0.01; Table 1).

**Table 1:** Correlation coefficients of myostatin and visceral fat area; glucose; insulin; HOMA-IR; BDNF; irisin and valine among ALL participants: WBC-CON and WBC-EXP group recorded before and after whole procedure.

	Visceral Fat Area (cm^−2^)			Glucose (mg∙dL^−1^)			Insulin (µU∙mL^−1^)			HOMA-IR			BDNF (ng∙mL^−1^)			Irisin (ng∙mL^−1^)			Valine (µmol∙L^−1^)		
	ALL	WBC-CON	WBC-EXP	ALL	WBC-CON	WBC-EXP	ALL	WBC-CON	WBC-EXP	ALL	WBC-CON	WBC-EXP	ALL	WBC-CON	WBC-EXP	ALL	WBC-CON	WBC-EXP	ALL	WBC-CON	WBC-EXP
**Myostatin **(**ng∙mL**^**−1**^)																					
Before	0.70*	0.40	0.57*	0.69*	0.41	0.59*	0.46*	0.16	0.68*	0.53*	0.31	0.70*	− 0.52*	− 0.19	− 0.65*	0.65*	0.66*	0.66*	0.06	0.07	− 0.14
After	0.11	0.10	0.06	0.08	0.38	− 0.05	0.34*	− 0.34	− 0.33	− 0.31	− 0.23	− 0.33	− 0.21	− 0.01	− 0.23	− 0.09	− 0.12	− 0.09	0.21	− 0.21	0.60*
*p*	**0.01**	0.48	**0.04**	**0.00**	0.94	**0.02**	0.57	0.26	**0.00**	**0.00**	0.23	**0.00**	0.15	0.69	**0.05**	**0.00**	**0.05**	**0.01**	0.54	0.53	**0.01**

Values are Spearman correlation; *statistically significant correlations; *p-*difference between the correlations; *p* < 0.05; Statistically significant differences between correlations are bold.

### Effects of a single session of WBC

In our assessment, we considered the analysis of blood samples collected before and 1hour after the first (acute) and the last (chronic) WBC exposure.

### Changes in resposne to the first session of WBC

**Figure 3:**
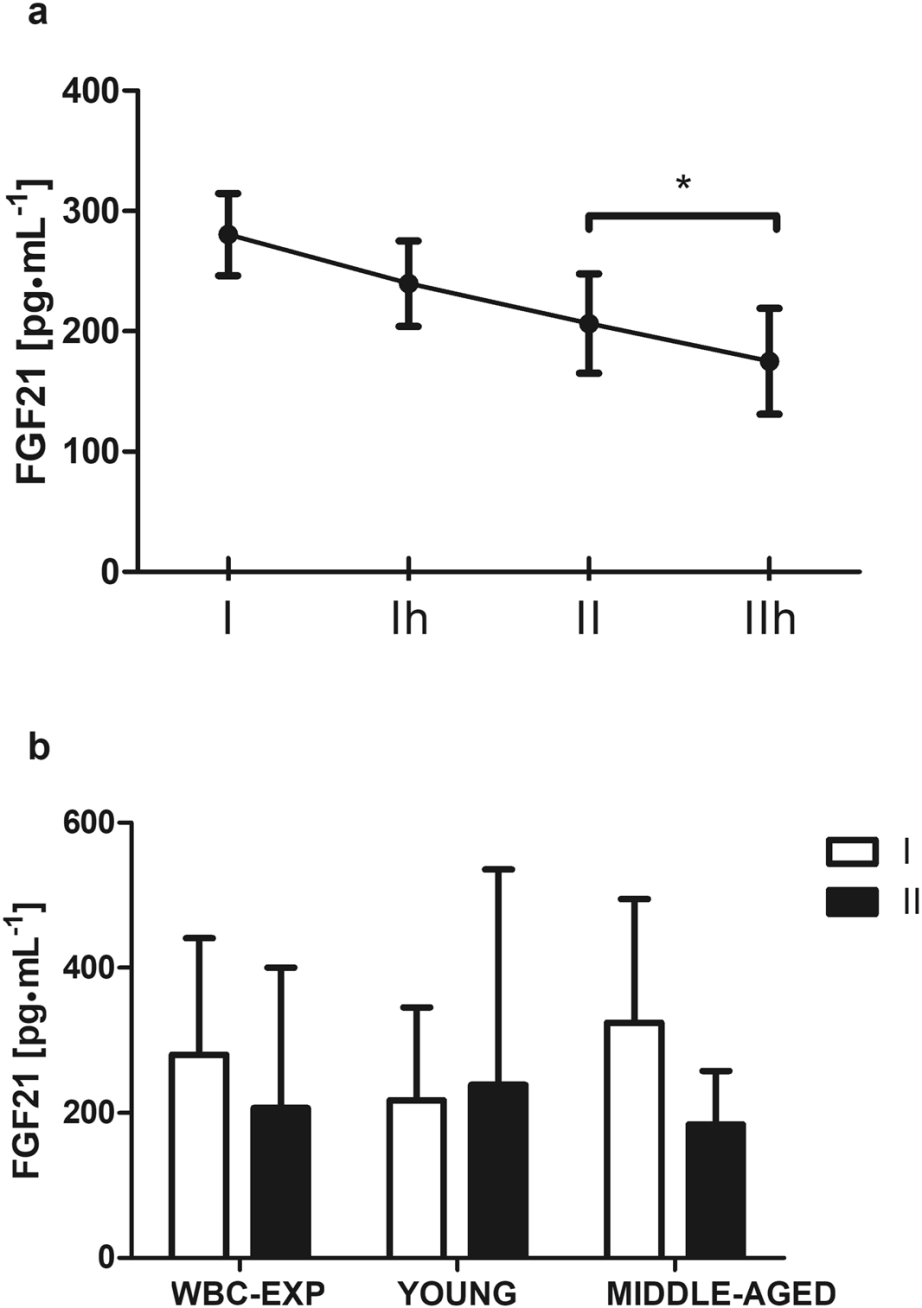
Changes in the concentration of FGF21 (data are presented as mean ± SEM) recorded: (**a**) at each point of blood collection: (I) before WBC, (Ih) 1 h after the first WBC, (II) before the last WBC and (IIh) 1 h after the last WBC; (**b**) in the WBC-EXP group with age-dependent changes before the first (I) and the last (II) session of WBC. *p < 0.05 significant differences between time point measurements.

The effect of the first WBC session on myokines concentrations is presented in Fig. 1. Irisin (*p* = 0.02) and myostatin (*p* = 0.03) concentrations increased significantly in the WBC-EXP group. This was not the case for BDNF concentration. However, when considering the age groups, the first WBC session resulted in a pronounced drop of BDNF and a significant increase of irisin (*p* = 0.01) concentration in YG participants, but not in MG individuals (Fig. 1a,c). A single WBC exposure also decreased FGF21 level in the WBC-EXP group (from 280.4 ± 160.5 to 239.7 ± 166.6 pg∙mL^-1^, *p* = 0.07; Fig. 3a). Changes in AA profile’s in response to a single session of WBC are presented in Table 2. Elevated levels of alanine, isoleucine, tryptophan, lysine, tyrosine, phenylalanine, methionine, arginine and threonine were recorded. The effect size expressed by Cohen’s d value ranged from medium to large.

**Table 2:** The effect of single session of the whole-body cryotherapy on amino acid profile.

	First session				Last session			
	Before	1 h post	*p-* value	Cohen’s-d	Before	1 h post	*p-* value	Cohen’s-d
**AA’s- the most important gluconeogenic precursors**								
Alanine (µmol∙L^−1^)	115.6 ± 26.2	147.2 ± 37.2*	**0.00**	0.79	116.4 ± 23.9	144.2 ± 43.1*	**0.00**	0.70
Glutamine (µmol∙L^−1^)	251.1 ± 57.5	265.4 ± 51.5	0.32	0.24	265.5 ± 62.4	267.5 ± 46.8	0.27	0.27
**AA’s- after deamination form keto acid like acetyl-CoA**								
Isoleucine (µmol∙L^−1^)	84.4 ± 30.6	117.4 ± 47.7*	**0.00**	0.86	96.5 ± 32.3	88.2 ± 26.5	0.33	0.11
Leucine (µmol∙L^−1^)	112.9 ± 70.3	115.5 ± 43.9	0.35	0.04	92.3 ± 30.7	157.8 ± 67.8*	**0.00**	0.53
Tryptophan (µmol∙L^−1^)	38.1 ± 11.5	45.5 ± 15.1*	**0.00**	0.94	35.1 ± 11.1	36.4 ± 8.7	0.53	0.17
Lysine (µmol∙L^−1^)	68.4 ± 13.7	85.6 ± 23.7*	**0.01**	0.67	71.9 ± 15.1	84.4 ± 16.8*	**0.02**	0.77
**AA’s- after deamination form keto acid like fumarate**								
Valine (µmol∙L^−1^)	97.8 ± 26.1	111.7 ± 31.5	0.09	0.39	88.7 ± 21.9	119.2 ± 27.7*	**0.00**	0.75
Asparagine (µmol∙L^−1^)	41.3 ± 12.7	45.9 ± 12.4	0.25	0.26	37.1 ± 6.2	45.2 ± 8.2*	**0.00**	0.27
Aspartic acid (µmol∙L^−1^)	3.6 ± 1.5	3.7 ± 2.2	0.91	0.05	4.7 ± 2.7	4.3 ± 1.6	0.55	0.39
Tyrosine (µmol∙L^−1^)	56.9 ± 12.4	78.1 ± 24.3*	**0.00**	0.88	67.1 ± 21. 5	69.1 ± 18.6	0.56	0.87
Phenylalanine (µmol∙L^−1^)	45.7 ± 10.0	63.0 ± 26.2*	**0.00**	0.72	49.8 ± 22.6	51.9 ± 20.4	0.32	0.33
**AA’s- after deamination form keto acid like alpha-ketoglutarate**								
Glycine (µmol∙L^−1^)	121.1 ± 34.0	120.9 ± 44.4	0.99	0.00	143.0 ± 47.6	110.1 ± 30.9*	**0.01**	0.26
Glutamic acid (µmol∙L^−1^)	19.3 ± 9.8	21.1 ± 9.7	0.51	0.17	18.1 ± 7.7	21.6 ± 8.3	0.11	0.19
Proline (µmol∙L^−1^)	111.5 ± 33.3	126.1 ± 30.0	0.05	0.42	110.3 ± 27.5	138.9 ± 29.5*	**0.00**	0.64
Methionine (µmol∙L^−1^)	14.8 ± 5.8	19.1 ± 9.8*	**0.04**	0.55	14.9 ± 4.6	15.1 ± 6.8	0.62	0.05
Histidine (µmol∙L^−1^)	55.9 ± 15.8	57.7 ± 15.0	0.68	0.09	61.5 ± 14.5	60.4 ± 7.7	0.64	0.32
Arginine (µmol∙L^−1^)	52.2 ± 8.1	66.7 ± 16.8*	**0.00**	0.82	55.8 ± 12.1	61.8 ± 12.8	0.13	0.66
**AA’s- after deamination form keto acid like pyruvate**								
Serine (µmol∙L^−1^)	89.3 ± 22.4	97.4 ± 36.5	0.61	0.23	87.1 ± 20.9	87.6 ± 30.4	0.91	0.05
Threonine (µmol∙L^−1^)	40.0 ± 9.7	46.9 ± 13.4*	**0.01**	0.55	41.1 ± 12.5	39.5 ± 9.5	0.47	0.04

Data are presented as mean ± SD; AAs- amino acids; *statistically significant difference (before vs 1 h); Statistically significant differences are bold; Cohen’s d- effect size: > 0.2 small, > 0.5-medium, > 0.8-large.

### Changes in resposne to the last session of WBC

Before the last WBC session, the circulating level of irisin remained elevated in YG (*p* = 0.01) but not MG subjects (Fig. 1c). Additionally, in MG individuals, the concentration of irisin correlated significantly with PBF% (r = 0.58, *p* < 0.01; supplementary Table S2). In YG subjects, a positive relationship between skeletal muscle mass (SMM) and irisin concentration was observed 1 h after the last exposure (r = 0.78, *p* < 0.01; supplementary Table S2). Blood analysis of the last WBC session revealed a continued decrease of FGF21 (*p* < 0.01; Fig. 3a). Adiponectin also tended towards a decrease (*p* = 0.04) contrary to the effect observed after the chronic WBC exposure (Fig. 1d). The Cohen’s d effect size was large for both proteins (1.08 for adiponectin and 1.12 for FGF21). The last WBC session affected circulating levels of alanine, leucine, lysine, valine, asparagine, glycine and proline, which increased significantly with the exception of glycine concentration, which declined (*p* = 0.01; Table 2). Moreover, at this time point, the level of alanine was significantly higher in MG subjects compared to YG individuals (163.5 ± 44.9 vs 116.3 ± 19.3 µmol∙L^−1^, respectively, *p* = 0.05). The Cohen’s d effect size for the AA’s was medium (> 0.5 but < 0.8), except for asparagine and glycine, where the observed change was small (0.27 and 0.26, respectively).

### Effects of chronic WBC

Our assessment of the effect of chronic WBC is based on the analysis of blood samples collected at rest before the first and the last exposure (completing nine sessions, before 10th session). A comparison of PBF% before and after chronic WBC exposure showed a reduction in the WBC-EXP group (19.3 ± 6.1 to 18.8 ± 6.0%, *p* = 0.03,${\eta }_{p}^{2}=$ 0.14). Chronic WBC exposure also resulted in a reduction of VFA (88.74 ± 40.39 to 84.41 ± 39.56 cm^2^, *p* = 0.03, ${\eta }_{p}^{2}=$ 0.13). Changes in glucose homeostasis indicators (glucose, insulin, HOMA indicators) and the lipid profile from the initial to the final WBC session are presented in Table 3. Fasting glucose level significantly decreased (*p* = 0.04, ${\eta }_{p}^{2}=$ 0.13), whereas the lipid profile was not affected. Additionally, a significant reduction of insulin (from 9.0 ± 2.1 to 6.9 ± 2.1 µmol∙L^−1^, *p* = 0.01, ${\eta }_{p}^{2}=$ 0.28) and HOMA-IR (from 1.21 ± 0.3 to 0.92 ± 0.3, *p* = 0.01, ${\eta }_{p}^{2}=$ 0.28) was recorded only in MG subjects (Supplementary Table S1). In the WBC-EXP group, HOMA-S increased by 19.6% compared to the baseline for all participants (*p* = 0.08). HOMA-B increased significantly only in YG individuals (from 71.6 ± 13.1 to 90.4 ± 21.4%, *p* = 0.01, ${\eta }_{p}^{2}=$ 0.3; Supplementary Table S1).

**Table 3:** The effect of chronic whole-body cryotherapy on lipid profile and glucose homeostasis indicators among WBC-EXP (n = 22) and WBC-CON (n = 13).

	WBC-EXP		WBC-CON		ANOVA	
	Before	After	Before	After	*p*	${\eta }_{p}^{2}$
Total cholesterol (mg∙dL^-1^)	191.8 ± 34.6	173.4 ± 31.3	183.2 ± 35.2	157.2 ± 15.5	0.08	0.02
HDL (mg∙dL^-1^)	55.5 ± 13.4	55.7 ± 16.5	56.7 ± 11.2	54.2 ± 7.7	0.38	0.02
LDL (mg∙dL^-1^)	110.3 ± 29.0	96.8 ± 32.8	106.3 ± 27.7	83.3 ± 16.3	0.36	0.03
Triglycerides (mg∙dL^-1^)	130.0 ± 68.3	104.4 ± 18.4	100.9 ± 57.1	98.9 ± 29.2	0.94	0.05
Glucose (mg∙dL^-1^)	102.1 ± 9.3	93.3 ± 10.6*	94.2 ± 6.2	89.4 ± 5.8*	**0.04**	**0.13**
Insulin (µmol∙L^-1^)	7.9 ± 2.6	6.7 ± 2.4	7.7 ± 2.9	7.0 ± 2.0	0.53	0.01
HOMA-S (%)	109.1 ± 49.7	130.1 ± 53.4	114.2 ± 50.2	119.5 ± 37.4	0.08	0.06
HOMA-B (%)	74.4 ± 13.9	81.7 ± 21.7	87.1 ± 25.4	91.1 ± 21.2	0.08	0.01
HOMA-IR	1.1 ± 0.4	0.9 ± 0.3	1.0 ± 0.4	0.9 ± 0.3	0.40	0.06

Data are presented as mean ± SD; *statistically significant difference between before and after measurements in the group, p < 0.05; ${\eta }_{p}^{2}$- effect sizes (partial eta squared): ≥ 0.01 small, ≥ 0.06 medium and ≥ 0.14 large effect; HDL: high density lipoprotein; LDL: low density lipoprotein; HOMA: The Homeostasis Model Assessment estimates: HOMA-B- β-cell function; HOMA-S: insulin sensitivity as percentages of a normal reference population and HOMA-IR: insulin resistance.

Statistically significant group x time interaction are bold.

Table 4 presents changes in biochemical markers and AA profile’s recorded at baseline and in blood collected before the last WBC session. Ulike following acute WBC exposure, chronic WBC did not affect BDNF. The elevated level of irisin induced by the first cryosession was maintained among YG participants (*p* = 0.04). FGF21 concentration continued to drop throughout the intervention (baseline WBC *p* = 0.57 vs final WBC session *p* < 0.01; Fig. 3a,b). Chronic WBC exposure was also accompanied by a significant increase of adiponectin (46.8%, *p* = 0.05, ${\eta }_{p}^{2}=$ 0.09) in comparison to the WBC-CON group. Further, chronic WBC caused a decline in the circulating myostatin concentration but only in MG subjects (-30%, *p* < 0.01; effect size was equal 0.58; Fig. 1b). The opposite- upward trend was noted in the whole WBC-CON group. Interestingly, chronic WBC exposure blunted the difference in myostatin concentration recorded at baseline between YG and MG subjects.

**Table 4:** The effect of chronic whole-body cryotherapy on biochemical indicators and amino acid profile among WBC-EXP (n = 22) and WBC-CON (n = 13).

	WBC-EXP		WBC-CON		ANOVA	
	Before	After	Before	After	*p*	${\eta }_{p}^{2}$
BDNF (ng∙mL^-1^)	16.6 ± 7.0	17.2 ± 7.2	19.2 ± 5.5	17.3 ± 5.1	0.24	0.04
Myostatin (ng∙mL^-1^)	34.5 ± 9.8#	29.2 ± 6.7^**+**^	25.3 ± 6.5	28.4 ± 7.7	**0.02**	**0.16**
Irisin (ng∙mL^-1^)	9.2 ± 5.4	12.0 ± 8.4	6.9 ± 2.7	9.0 ± 2.7*	**0.03**	**0.11**
Adiponectin (µg∙mL^-1^)	12.5 ± 1.9	18.4 ± 5.7#	12.2 ± 3.5	12.6 ± 4.0	**0.05**	**0.09**
FGF21 (pg∙mL^-1^)	280.4 ± 160.5	206.5 ± 193.6	246.4 ± 149.0	184.2 ± 161.6	0.06	0.01
°Alanine (µmol∙L^-1^)	115.6 ± 26.2	116.4 ± 23.9	124.2 ± 31.9	121.0 ± 31.0	0.92	0.01
°Glutamine (µmol∙L^-1^)	251.1 ± 57.5	265.5 ± 62.4	282.1 ± 62.4	301.5 ± 33.3	0.08	0.01
■Isoleucine (µmol∙L^-1^)	84.4 ± 30.6	96.5 ± 32.3	100.9 ± 60.6	102.5 ± 37.2	0.17	0.01
■Leucine (µmol∙L^-1^)	112.9 ± 70.3	92.3 ± 30.7	85.3 ± 21.2	106.9 ± 36.4	0.81	0.09
■Tryptophan (µmol∙L^-1^)	38.1 ± 11.5	35.1 ± 11.1	36.1 ± 10.9	37.2 ± 14.5	0.22	0.04
■Lysine (µmol∙L^-1^)	68.4 ± 13.7	71.9 ± 15.1	83.9 ± 27.2	78.1 ± 18.8	0.11	0.03
▲Valine (µmol∙L^-1^)	97.8 ± 26.1	88.7 ± 21.9#	91.6 ± 22.5	122.9 ± 25.1*	**0.00**	**0.28**
▲Asparagine (µmol∙L^-1^)	41.3 ± 12.7	37.1 ± 6.2#	41.4 ± 12.2	49.6 ± 8.2	**0.01**	**0.17**
▲Aspartic acid (µmol∙L^-1^)	3.6 ± 1.5	4.7 ± 2.7	4.9 ± 1.3	6.6 ± 2.5	0.59	0.00
▲Tyrosine (µmol∙L^-1^)	56.9 ± 12.4	67.1 ± 21.5	65.7 ± 16.9	66.9 ± 24.5	0.19	0.05
▲Phenylalanine (µmol∙L^-1^)	45.7 ± 10.0	49.8 ± 22.6	48.6 ± 13.3	51.3 ± 11.0	0.42	0.01
♦Glycine (µmol∙L^-1^)	121.1 ± 34.0	143.0 ± 47.6	118.1 ± 30.6	144.0 ± 46.0	0.80	0.01
♦Glutamic acid (µmol∙L^-1^)	19.3 ± 9.8	18.1 ± 7.7	14.2 ± 8.4	22.1 ± 8.3	0.18	0.06
♦Proline (µmol∙L^-1^)	111.5 ± 33.3	110.3 ± 27.5	108.8 ± 47.2	130.4 ± 47.7	0.27	0.09
♦Methionine (µmol∙L^-1^)	14.8 ± 5.8	14.4 ± 4.6	13.9 ± 3.9	13.5 ± 6.3	0.74	0.01
♦Histidine (µmol∙L^-1^)	55.9 ± 15.8	61.6 ± 14.5	49.9 ± 10.6	62.3 ± 13.9	0.16	0.06
♦Arginine (µmol∙L^-1^)	52.2 ± 8.1	55.8 ± 12.1	59.4 ± 15.2	57.2 ± 10.9	0.68	0.03
●Serine (µmol∙L^-1^)	89.3 ± 22.4	87.1 ± 20.9	84.7 ± 25.0	89.1 ± 22.8	0.45	0.02
●Threonine (µmol∙L^-1^)	40.0 ± 9.7	41.1 ± 12.5	39.5 ± 12.9	39.9 ± 10.3	0.94	0.05

Data are presented as mean ± SD; *statistically significant difference between before and after measurements in the group; # statistically significant difference between groups at a time point, ^+^ statistically significant difference between before and after measurements MG from WBC-EXP vs WBC-CON *p* < 0.05;${\eta }_{p}^{2}$- effect sizes (partial eta squared): ≥ 0.01 small, ≥ 0.06 medium and ≥ 0.14 large effect; Amino acids after deamination form keto acid like: °the most important gluconeogenic precursors in liver ; ■acetyl-CoA; ▲fumarate; ♦alpha-ketoglutarate; ●pyruvate, which are further metabolized in gluconeogenesis process in the Krebs-cycle.

Statistically significant group x time interaction are bold.

Regarding changes in AA profile’s, the concentrations of valine (*p* < 0.01) and asparagine (*p* < 0.01) were significantly lower in the WBC-EXP than in the WBC-CON upon the last session of WBC. At this point in time, a positive correlation between valine and myostatin was recorded in the WBC-EXP group (r = 0.60; Table 1). The remaining AA’s were not affected by the intervention (Table 4).

## Discussion

Our results demonstrate that chronic WBC exposure had a positive effect on glucose homeostasis in normoglycemic participants. This exposure caused a significant decrease of blood glucose concentration and ameliorated most of the measured indicators of glucose homeostasis. There was also a significant reduction of glucose, evident in the WBC-CON, but still the decrease noted among experimental WBC-EXP group was two-fold higher compared to the WBC-CON group. Significant reductions of insulin and HOMA-IR values were particularly visible among MG participants subject to WBC. The level of these factors was elevated at baseline compared to YG subjects, thus the effect of the intervention in MG participants was more pronounced. Beneficial changes in glucose homeostasis may be connected with the activation of the hypothalamic–pituitary–adrenal axis and the sympathetic nervous system^27^. A recently published paper by Yoneshiro et al. (2019) revealed that cold exposure significantly reduced plasma concentrations of valine, leucine and isoleucine. The authors relied on plasma metabolomics in obese mice and measured the acitivity of BAT, which displayed the highest valine oxidation in cold exposure, relative to other metabolic organs. In a follow up study, these observations were also verified on humans^37^. Therefore, a WBC induced reduction in glucose concentration evident in our study may have modified the activity of white as well as BAT resulting in reduced PBF% and VFA.

To the best of our knowledge, our study is the first to assess strictly the effect of WBC on blood concentrations of AA’s in men. Previously, only one study demonstrated a significant drop of tryptophan and valine after 10 sessions of WBC combined with volleyball training^38^. In the present study, we assessed AA concentrations based on their role in glucose homeostasis. Similar to previous research in animal models (Yoneshiro et al. 2019), we noted a drop of valine in the WBC-EXP group following chronic WBC exposure compared to the WBC-CON group. In the present study, the observed decrease in valine following WBC likely occurred either because WBC could have induced the conversion of valine to β-aminoisobutyric acid, which is a myokine involved in the browning of fat^39^. Or because cold treatment stimulated the activity of mitochondrial BCAA enzymes such as the branched-chain α-keto acid dehydrogenase complex in the white adipose tissue^37^. Hence, the observed decrease of valine could have been associated with a statistically significant decrease of glucose concentration accompanied by the downward trend of insulin and HOMA-IR recorded in the MG part of the WBC-EXP group. At the same time, we noted a significant drop of VFA in the whole WBC-EXP group. This reduction in the amount of VFA might have had also a diminishing effect on its endocronical action.

Circulating concentrations of almost half of the AA’s increased significantly 1 h after the first WBC session. It is possible that, at this point in time, the protein breakdown peaked and AA’s were released into the bloodstream. This hypothesis is supported by the fact that this trend of change was also observed 1 h after the last session of WBC. Among all of the AA’s only changes in alanine followed the same trend in response to first as well last session of WBC. Increased metabolism of BCAA in skeletal muscle during WBC, which is manifested by a decrease in serum valine, may lead to increased alanine formation^40^. Thus, alanine can be transported to the liver to act as a substrate in the gluconeogenesis process. Nevertheless, chronic WBC exposure did not affect alanine expression in the present study. Meanwhile, only concentrations of valine and asparagine were reduced following the chronic WBC. This response might be beneficial in IR individuals because a previous study revealed that high concentrations of BCAA, phenylalanine, tyrosine, alanine, ornithine and lysine were associated with an increased risk of T2DM^41^. Further, valine and asparagine belong to an AAs signature associated with T2DM risk and progression. Particularly, while increased valine levels together with isoleucine and leucine predict T2DM risk, increased asparagine is associated with a progression of diabetes (along with aspartic acid, glutamine and glutamate)^42^. It is possible to hypothesize that if chronic WBC exposure is capable of reducing AA expression in normoglycemic participants, a similar response in hyperglycemic individuals would be beneficial. Therefore, the beneficial effects of WBC on metabolism can be marked by the improved AA profile.

Together with the improvement of AA profile’s, we noted a drop of myostatin among MG participans of WBC-EXP group. In addition to regulating muscle cell growth, myostatin has been shown to inhibit glucose uptake^43^, which suggests that it may contribute to systemic IR. Elevated myostatin levels were registered in pathological conditions characteristic of the metabolic deregulations such as obesity, T2DM and aging^44^. Our results are consistent with those findings. We observed a significant correlation between myostatin and most of the glucose homeostasis indicators at baseline. Also, at baseline, MG exhibited higher concentrations of myostatin than YG ones. These data are consistent with the findings of Yarasheski et al. (2002), who reported elevated serum myostatin in advance-aged men compared with younger subjects^45^. Interestingly, following chronic WBC exposure, serum myostatin dropped in MG subjects to the level recorded in their YG counterparts. This response could be beneficial, particularly in MG individuals at risk of hyperglycaemia.

In addition to skeletal muscles, BAT is a significant source of myostatin^46,47^. Cold exposure has been shown to up-regulate the transcription of interferon regulatory factor 4 (IRF4) in BAT, leading to inhibition of the myostatin expression^46^. In comparison, heat exposure (30 °C) or loss of IRF4 function have been reported to result in an elevation of serum myostatin^46^. Report of Kong et al. (2018) revealed that BAT can secrete significant amounts of myostatin into the blood; therefore, cold treatment can be expected to inhibit the secretion of myostatin from BAT^46^. In contrast, Zak et al. (2018) previously observed that the synthesis of myostatin in skeletal muscles was not sensitive to temperature^48^. Based on these reports, our data suggest that the effects of WBC were related to its impact on BAT rather than skeletal muscles. Importantly, the changes observed in serum myostatin were age-dependent. Sliwicka et al. (2020) observed that shifts in myostatin induced by cold treatment and/or physical exercise were only temporary in young men and returned to the baseline level within 24 h following cryotherapy/exercise^27,49^. In the present study, changes in myostatin concentration were more pronounced in MG participants, subject to chronic WBC, after which they exhibited serum myostatin at the level observed in YG subjects at baseline.

Myostatin acts through the inhibition of Akt kinase, which can lead to the activation of FOXO3a, a transcription factor that induces the expression of atrogin-1 gene encoding for a protein strongly linked to muscle atrophy. Thus, reducing the expression of myostatin through chronic WBC exposure can possibly improve the uptake of AA’s in MG individuals, and indirectly, ameliorate insulin sensitivity^50^.

The improvement of glucose homeostasis was accompanied by changes in FGF21 concentration in the present study. These results are partly comparable to those reported by Shabkhiz et al. (2020)^51^. They observed a decrease of FGF21 and myostatin, which suppressed IR in elderly men after 12-weeks of resistance training^51^. On the other hand, elevated circulating levels of FGF21 have been reported in the elderly^52^ and in T2DM patients^53^. In the present study MG participants demonstrated an elevated concentration of FGF21 compared to YG counterparts at baseline. WBC induced a drop of FGF21 among all participants, however, these changes were age- dependent. Previously, Hollstein aet al. (2020) also observed a decrease in plasma FGF21 after a long-term cooling session (24 h inside a calorimeter at 19.0 ± 0.3 °C) in overweight and obese participants^54^. Others have reported conflicting effects on FGF21; with one study reporting an increased secretion of FGF21 (12 h exposure to 24 °C or 19 °C in a whole-room indirect calorimeter)^55^, while a second study reported a decrease in FGF21 (cooling vest ~ 14.5 °C for 1-2 h)^28^. The disparity in the aforementioned results could be a result of the cooling protocols which differed significantly from the extremely low temperature applied in our WBC intervention. Furthermore, the variations in FGF21 concentrations can also be attributed to the different time points at which FGF21 was measured particulary because the circadian rhythm modulates a nightly increase and daily decrease in FGF21^56^. In order to standardize our data collection, and in an attempt to mitigate the impact of circadian rhythm on FGF21 concentration as well as other tested markers^57,58^, we collected blood samples at the same time of day on each day of our data collection.

Together with FGF21, irisin represents a link between myostatin and glucose metabolism^59^. Lee et al. (2014) demonstrated that both irisin and FGF21 are cold-modulated factors that participate in the regulation of glucose metabolism^30^. In the present study, we observed a significant increase in serum irisin 1 h after the first WBC session, particularly among YG participants. This effect was sustained throughout the study protocol. We also observed two-fold higher values of irisin at baseline in MG participants compared to YG. This observation is consistent with that of Huth et al. (2015), who found a positive correlation between irisin, age and obesity markers, which all correlated inversely with insulin sensitivity^60^. Changes in irisin concentration in response to WBC can be linked with the two sources of this protein: skeletal muscles^30^ and fat tissue^31,61^. The correlations recorded in WBC-EXP group in our study would suggest that the origin of irisin during cold exposure depended on body composition. In our previous study, we concluded that the effect of WBC on irisin concentration depended on participants’ physical fitness level^31^, thus men with a similar level of relative VO_2_max were recruited for this experiment. Chronic WBC caused significant increase in irisin concentration, but we did not observe any correlations of this change with body composition or fitness level.

It is worth noting that the elevated concentration of irisin at baseline was accompanied by a lower level of BDNF in MG participants, who were also characterized by higher adiposity compared to the YG individuals. This relationship was also confirmed by a statistically significant, inverse correlation between irisin and BDNF in the whole experimental group. BDNF is hypothesized to be a growth factor with a strong influence on peripheral metabolism, including fat oxidation and the subsequent effect on adipose tissue^62^. Krabble et al. (2007) noted low levels of circulating BDNF in individuals with both obesity and T2DM^63^. Moreover, Pedersen et al. (2009) observed an inverse correlation between plasma BDNF and glucose, which raises a possibility that high plasma glucose levels would negatively influence BDNF concentration^62^. Significantly higher glucose concentration among MG subjects recorded at baseline in our study, partly confirm these findings. BDNF did not change in response to the single and chronic exposure to WBC.

Chronic cold exposure caused an increase of the level of adiponectin in the WBC-EXP group, yet no significant changes were observed in the WBC-CON group. Similarly, Imbeault et al. (2009) observed an increase in adiponectin levels in young healthy men during a 2 h period of cold exposure (both 4 °C and 10 °C) which was inhibited by glucose ingestion^64^. Adiponectin is considered a marker of systemic insulin sensitivity^65^. In the present study, the elevation in adiponectin concentration was accompanied by a decrease of insulin and glucose in the WBC-EXP group at the conclusion of the tenth WBC exposure. Despite the differences in PBF% among YG and MG participants, the mean change of adipokine during WBC exposure did not differ significantly between these two cohorts. Nevertheless, a trend towards an increase in adiponectin among MG individuals compared with YG individuals (mean increase of 54.6% vs 35.5%, respectively) was noticed. Adiponectin is one of the most abundant adipokines secreted by adipocytes^65^. Our findings suggest that the amount of body adipose tissue may have affected the relative increase of circulating adiponectin during WBC.

**Figure 4:**
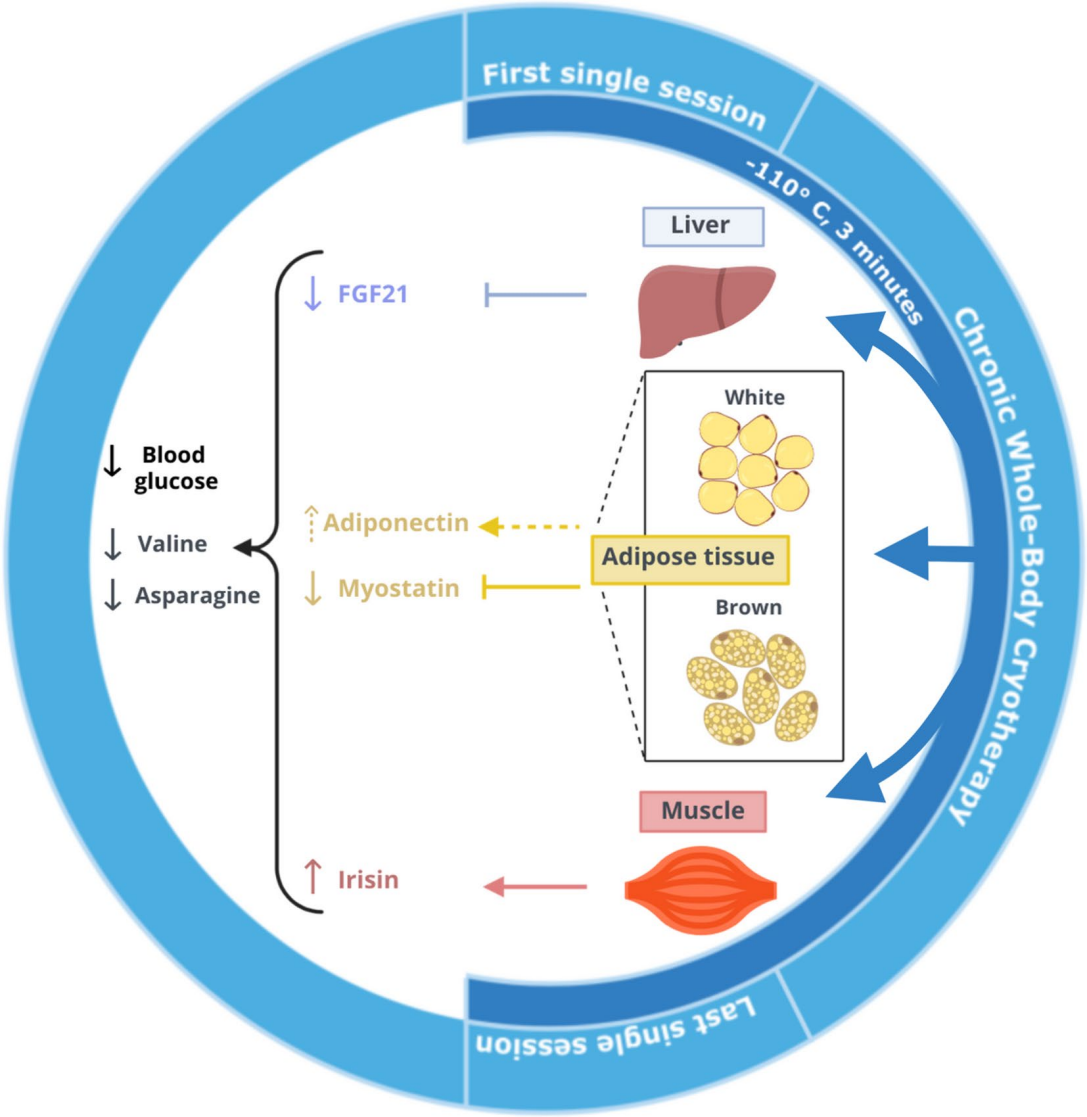
Graphical conclusion–proposed interpretation of the findings. WBC inhibited FGF21 in the liver, triggered skeletal muscle mass shivering, which lead to an increased release of irisin, and stimulated brown fat tissue to reduce myostatin and white fat tissue to release adiponectin.

To conclude, the both the acute and chronic WBC protocol led to an improvement in glucose homeostasis indicators together with a reduction of valine and asparagine (Fig. 4). These changes were accompanied by a decline of serum myostatin concentration. This effect was more pronounced amongst the MG participants. Our intervention is not without limitations. We did not perform fat or muscle tissue biopsy, which means that we cannot clearly determine the source of the indicators observed in the blood. Further research could address these limitations, in particular determine the longevity of the WBC-induced effects on reduce myostatin level, changes in blood AA profile, improvement of glucose homeostasis and explore other factors modulating these effects. Overall, our results support the use of WBC to induce at least a short-term improvement in the metabolic profile that may feed into more complex preventive strategies, including physical activity and eventually, pharmacologic interventions, against the risk of development of IR and T2DM.

## Methods

### Subjects

**Figure 5:**
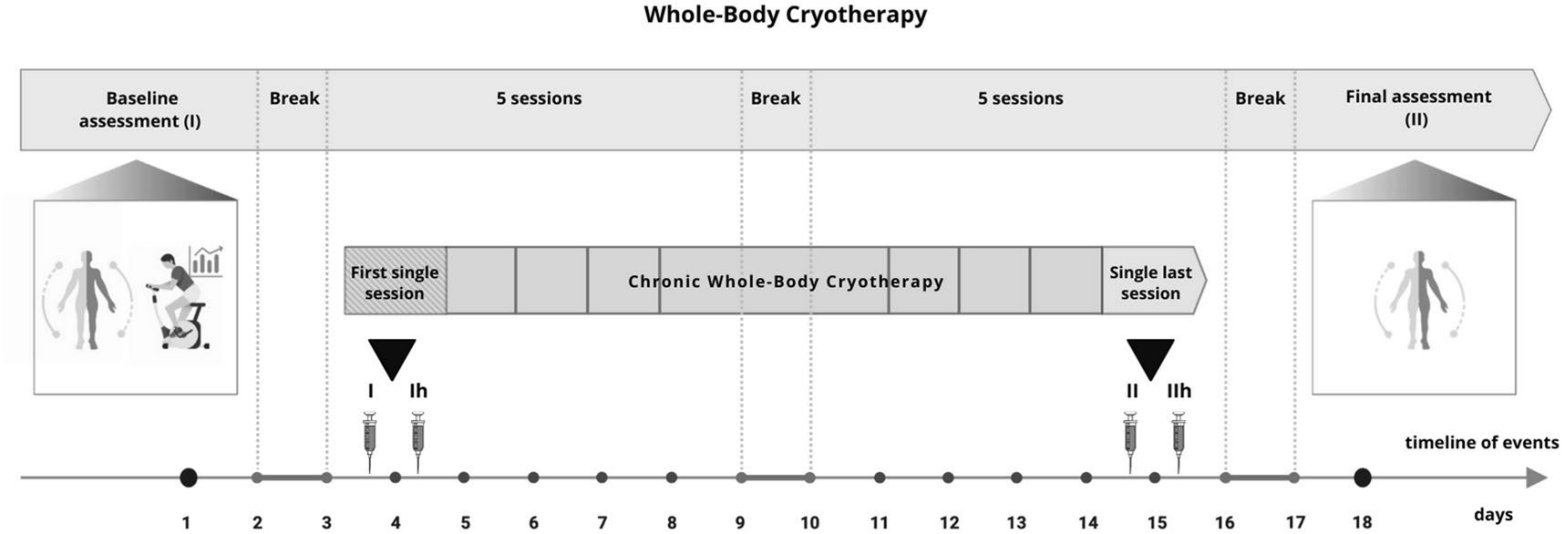
The experiment schedule. Blood collection: (I) before the first WBC session, (Ih) 1 h after the first WBC, (II) before the last WBC session and (IIh) 1 h after the last WBC session. Baseline assessment: body composition assessment and cardiorespiratory fitness measurement. Final assesment: body composition asesssement.

Thirty-five healthy, untrained, male participants, who had not experienced WBC in the previous 12 months, took part in the experiment. Prior to participation in the study, volunteers completed a medical screening in order to eliminate those with contraindications to cold exposure (e.g. cardiovascular disease, blood pressure > 160/100 mmHg, stroke or cold intolerance)^66^. Using an online software https://www.graphpad.com/quickcalcs/randMenu/), the participants were randomly assigned to either the experimental WBC group (WBC-EXP, n = 22; age = 40 ± 13.5 years; BMI = 26.1 ± 3.9 kg∙m^2^; PBF% = 19.3 ± 6.1%) or the control group (WBC-CON, n = 13; age = 30.1 ± 7.4 years; BMI = 23.5 ± 2.5 kg∙m^2^; PBF% = 17.2 ± 5.9%). The WBC-EXP group was further divided intoeither young (YG; n = 9; age = 28 ± 7 years) or middle-aged (MG; n = 13; age = 51 ± 3 years). Considering previous reports, which revealed that the effect of WBC on circulating myokines and adipokines was depended on participants’ cardiorespiratory fitness^36^, that the participants in the present study all had a similar relative VO_2_max (WBC-CON 47.4 ± 4.6 mL kg^-1^ min^-1^; WBC-EXP 46.5 ± 5.1 mL kg^-1^ min^-1^). The study protocol was approved by the Bioethical Committee of the Regional Medical Society in Gdansk KB-28/17 and was conducted in accordance with the Declaration of Helsinki. This experiment was conducted as an arm of the clinical trial registered in the ClinicalTrials.gov: NCT04375969 on 6 May 2020. A written, informed consent was obtained from all subjects. A schematic representation of the experimental protocol is presented in Fig. 5.

### Body composition assessment

Body mass and body composition, skeletal muscle mass, PBF% and VFA^67,68^ were estimated using a multi-frequency impedance analyser (In Body 720, Biospace, Korea). Measurements were taken on the first day of data collection and after the final session of WBC with the participants in a fasted state. During the measurement, subjects wore only shorts and remained barefoot. The impedance of segments of different body parts (trunk, arms and legs) was measured at six different frequencies (1, 5, 50, 250,500, and 1000 kHz) using an eight-polar tactile-electrode. This method can be used as a surrogate of dual-energy X-ray absorptiometry^69^ because of greater availability and smaller individual error produced by InBody analyzer, which makes it equally precise.

### Cardiorespiratory fitness measurement

In order to standarize the study group in terms of cardiorespiratory fitness, participants performed a graded cycle test on a cycle ergometer (884E Sprint Bike Monark, Sweden) to determine their VO_2_max. The test was conducted three days prior to the start of the first WBC exposure. The VO_2_max test began with a 5-min warm up at a workload of 1.5 W·kg^−1^ and a pedalling cadence of 60 rpm. The load increased progressively by 25 W·min^−1^ until an individual reached the point of volitional exhaustion. Pulmonary gas exchange was measured during the test (MetaMax 3B, Cortex, Germany)^36^. The highest value of relative oxygen uptake was taken into consideration when assigning experiment and control groups.

### Blood analysis and collection

Blood samples were taken on the first day of the WBC treatment (both prior to and 1 h after the first session) and on the last day of the final WBC session (also both prior to and 1 h after the last, 10th session). Samples (approx. 20 ml per person during each collection) were collected from the antecubital vein uding a needle into vacutainer tubes with K3EDTA (Becton, Dickinson & Co., Franklin Lakes, NJ, USA) for plasma analysis, and into vacutainer’s with sodium fluoride to estimate glucose concentration and SSTTM II Advance for serum analysis. Samples were centrifuged at 2000 g at 4 °C for 10 min and then stored at −80 °C.

Serum FGF21, myostatin and BDNF were determined by enzyme immunoassay methods using commercial kits (R&D Systems, USA; catalog no. DF2100, DGDF80 and DBD00, respectively) in accordance with manufacturer’s instructions. The detection limits were 8.69 pg∙mL^−1^ for FGF21, 2.25 pg∙mL^−1^ for myostatin and < 20 pg∙mL^−1^ for BDNF. The average intra-assay coefficient of variation (CV) was 3.5–3.9% for FGF21 and 5% for myostatin and BDNF. For myostatin measurements, samples were diluted in a 1:4 ratio (in 1 N HCL, 1.2 N NaOH/0.5 M HEPES and Calibrator Diluent RD5-26) prior to the analysis according to the manufacturer’s instruction.

Quantification of serum irisin and plasma adiponectin was determined via the enzyme immunoassay method using commercially available kits from Phoenix Pharmaceuticals Inc, USA (catalog no. EK 067–29 and EK- ADI-01, respectively) according to the manufacturer´s protocol. For irisin, intra-assay CV was 4–6% and inter-assay CV was 8–10%. For adiponectin intra-assay and inter-assay CV’s were < 10% and < 15% respectively, and detection sensitivity was 5.32 pg mL^−1^. AA profile was conducted based on the ion-pair reversed phase high performance liquid chromatography combined with the tandem mass spectrometry IP-RP HPLC–MS/MS (TSQ Vantage Thermo Scientific, USA). The procedure was executed following the protocol already described by Gmiat et al.^70^.

Glucose level was assessed using the Cobas 6000 analyser. To determine insulin concentration the immunoassay kit from DiaMetra (catalogue no DKO076, Perugia, Italy) was used. The intra-assay CV was ≤ 5% and the inter-assay CV was ≤ 10%. Homeostasis model assessments for insulin sensitivity (HOMA-S), β-cell function (HOMA-B) and insulin resistance (HOMA-IR) were obtained from paired fasting glucose and insulin levels using the updated software HOMA calculator, version 2.2.3, copyright by The University of Oxford (www.dtu.ox.ac.uk/homacalculator). Normal values are 100% for HOMA-S and HOMA-B and 1.0 for HOMA-IR^71^.

### Whole-body cryotherapy procedure

WBC sessions took place in a cryogenic chamber (Zimmer MedizinSysteme, Elecpol) at the Pomeranian Rheumatologic Centre in Sopot, Poland. The treatments were performed five days in a row, with a two-day rest period, followed by five more consecutive days, for a total of 10 sessions completed over two weeks (Fig. 5). Sessions took place at the same time of day (in the morning between 7:30 am and 8:00 am after a light breakfast). Each session was preceded by a 30-s adaptation in the chamber at − 60 °C. The cryotherapy exposure in the main chamber lasted 3 min at − 110 °C. Participants wore shorts, socks, gloves and a hat to protect their hands, feet and ears againts frostbite. According to the instructions, they moved slowly on a circle, changing direction of the motion every 1 minute^72^. Participants did not engage in any other recovery treatment, throughout the duration of the study.

### Statistical analysis

Statistical analyses were performed using a dedicated software package (Statistica 13.1 software, TIBCO Software, Palo Alto, California, USA). The sample size of the study group was predetermined using a power calculation in the software G ∗ power version 3.1.9.4^73^ (a priori repeated-measures within-between interaction, α = 0.05, 1-β = 0.95, r = 0.6, f = 0.25, ε = 1, with a further 20% surcharge due to the possibility of the participant not completing the experiment). Shapiro–Wilk tests were used to assess the homogeneity of dispersion from normal distribution. Brown–Forsythe test was used to evaluate the homogeneity of variance. To analyse the effect of a single cryotherapy session, we used paired tests for a homogenous sample. For a heterogeneous sample, Wilcoxon signed-rank test was used. In the second phase of analysis, for lipid profile, amino acid profile and glucose homeostasis indicators two (group: WBC-EXP, WBC-CON) x two (time: before and after 2 weeks) analyses of repeated measurements of variances (ANOVA) were calculated. In case of a significant group x time interaction, for homogenous results Tukey’s post hoc tests for unequal sample sizes were performed to identify significantly different results. For heterogeneous results, ANOVA Friedman’s test and Dunn-Bonferroni post-hoc test were used. The effect size (partial eta squared, ${\eta }_{p}^{2}$) was also calculated, with ${\eta }_{p}^{2}$≥0.01 indicating a small effect; ≥ 0.059 indicating a medium effect; and ≥ 0.138 indicating a large effect^74^. A similar analysis was done for age groups (YG and MG) in the WBC-EXP treatment group. Relationships between variables were evaluated using the Spearman correlation coefficient. Additionally, the effect size (Cohen’s d) was calculated, with d ≥ 0.2 indicating small effect; ≥ 0.5 indicating medium effect; and ≥ 0.8 indicating large effect. The level of significance was set at *p* < 0.05. In the descriptive analysis, data are reported as a mean ± standard deviation (SD).

## Supplementary Information

Supplementary Figures

Supplementary Tables
