# Supplementary figures and images for: Beneficial effects of whole-body cryotherapy on glucose homeostasis and amino acid profile are associated with a reduced myostatin serum concentration
Source: Sci Rep. 2021 Mar 29;11:7097. doi: 10.1038/s41598-021-86430-9 (PMC8007810; doi:10.1038/s41598-021-86430-9)

Supplementary Figure 1. Flow chart of the study participants.

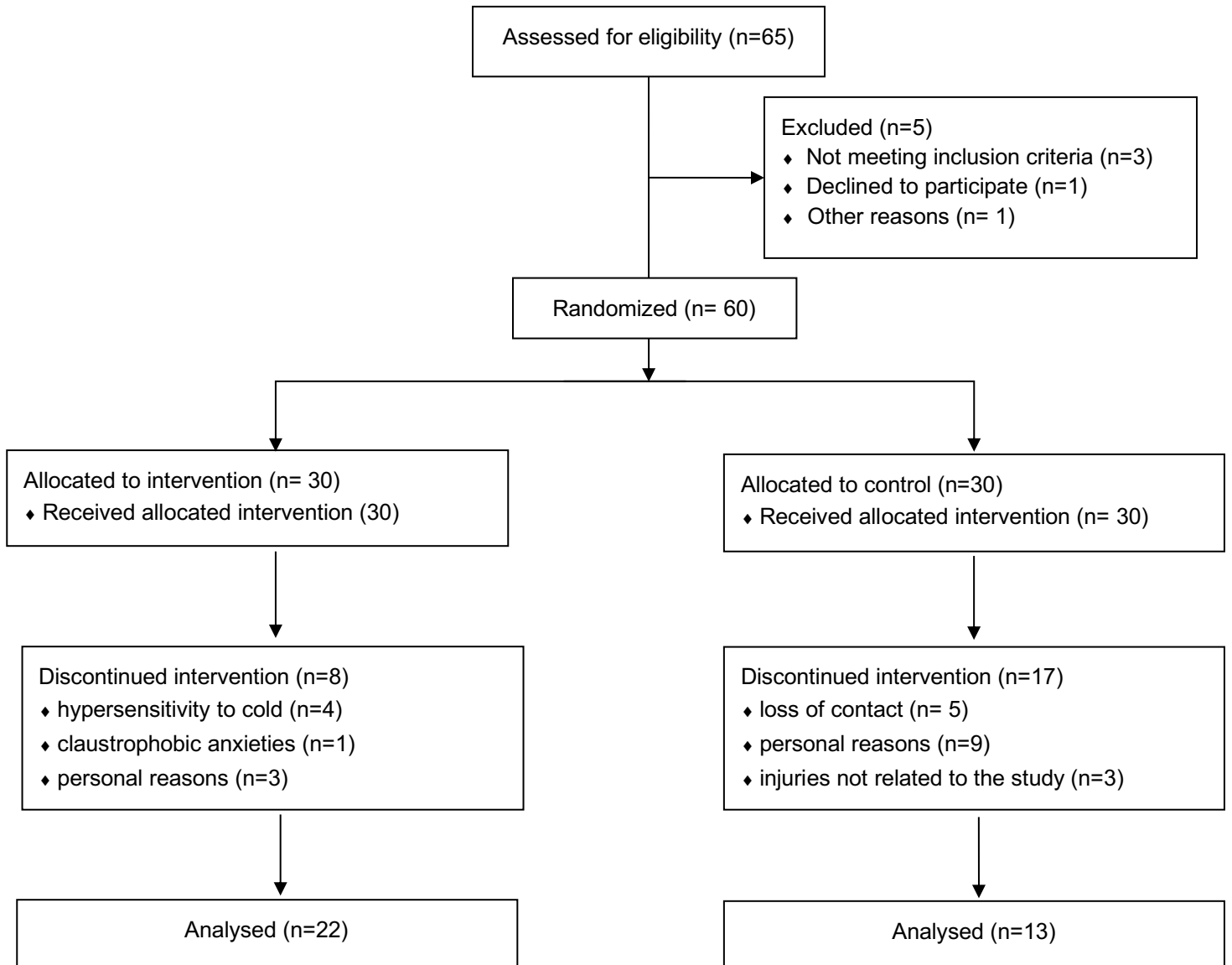

Supplement: Supplementary file 1 — Supplementary Figures [file 41598_2021_86430_MOESM1_ESM.pdf]
