# Supplementary material for: Beneficial effects of whole-body cryotherapy on glucose homeostasis and amino acid profile are associated with a reduced myostatin serum concentration
Source: Sci Rep. 2021 Mar 29;11:7097. doi: 10.1038/s41598-021-86430-9 (PMC8007810; doi:10.1038/s41598-021-86430-9)
Supplement: Supplementary file 2 — Supplementary Tables [file 41598_2021_86430_MOESM2_ESM.docx]

Supplementary Information

**The beneficial effects of whole-body cryotherapy on glucose homeostasis and amino-acid profile are associated with myostatin serum concentration**

Marta Kozłowska, Jakub Kortas, Małgorzata Żychowska, Jędrzej Antosiewicz, Klaudia Żuczek, Silvia Perego, Giovanni Lombardi, Ewa Ziemann

| Table S1. The effect of nine sessions of whole-body cryotherapy on lipid profile, glucose homeostasis and biochemical indicators among YG (n=9) compare to MG (n=13). | | | | | | | | | | | | | | | | | | | | | | | | | | | | | | |  |  |  |  |
| --- | --- | --- | --- | --- | --- | --- | --- | --- | --- | --- | --- | --- | --- | --- | --- | --- | --- | --- | --- | --- | --- | --- | --- | --- | --- | --- | --- | --- | --- | --- | --- | --- | --- | --- |
|  | | | YG | | | | | | | | | | | | | | | MG | | | | | | | | | ANOVA | | | | |  |  |  |
|  | | | before | | | | | | | | | after | | | | | | before | | | | | | after | | | *p* | | $\eta_{p}^{2}$ | | | | |  |
| Total cholesterol [mg∙dL^-1^] | | | | 173.4 | | | ± | 23.4# | | | 157.3 | | | ± | | 26.3 | | | 204.5 | | ± | 36.0 | 184.5 | | ± | 30.5 | | 0.75 | | 0.00 | | |  |  |
| HDL [mg∙dL^-1^] | | | | 49.6 | | | ± | 7.4 | | | 48.0 | | | ± | | 11.1 | | | 59.6 | | ± | 15.3 | 61.0 | | ± | 17.8 | | 0.09 | | 0.02 | | |  |  |
| LDL [mg∙dL^-1^] | | | | 100.7 | | | ± | 20.1 | | | 90.1 | | | ± | | 23.1 | | | 116.9 | | ± | 33.0 | 101.4 | | ± | 38.3 | | 0.72 | | 0.01 | | |  |  |
| Triglycerides [mg∙dL^-1^] | | | | 115.5 | | | ± | 55.8 | | | 95.9 | | | ± | | 19.3 | | | 140.1 | | ± | 76.3 | 110.4 | | ± | 15.9 | | 0.83 | | 0.00 | | |  |  |
| Glucose [mg∙dL^-1^] | | | | 95.7 | | | ± | 8.6# | | | 86.4 | | | ± | | 11.5 | | | 106.5 | | ± | 7.0 | 98.1 | | ± | 6.9 | | 0.07 | | 0.06 | | |  |  |
| Insulin [µU∙mL^-1^] | | | | 6.3 | | | ± | 2.5# | | | 6.4 | | | ± | | 2.8 | | | 9.0 | | ± | 2.1 | 7.0 | | ± | 2.1***** | | **0.01** | | 0.28 | | |  |  |
| HOMA-S [%] | | | | 141.6 | | | ± | 62.3# | | | 146.0 | | | ± | | 67.1 | | | 86.6 | | ± | 20.0 | 119.0 | | ± | 40.9 | | 0.14 | | 0.09 | | |  |  |
| HOMA-B [%] | | | | 71.6 | | | ± | 13.1 | | | 90.4 | | | ± | | 21.4***** | | | 76.4 | | ± | 14.7 | 75.7 | | ± | 20.5 | | **0.01** | | 0.3 | | |  |  |
| HOMA-IR | | | | 0.8 | | | ± | 0.4 | | | 0.8 | | | ± | | 0.4 | | | 1.2 | | ± | 0.3 | 0.9 | | ± | 0.3***** | | **0.01** | | 0.28 | | |  |  |
| BDNF [ng∙mL^-1^] | | | | 21.9 | | | ± | 5.7# | | | 22.4 | | | ± | | 5.4 | | | 13.0 | | ± | 5.4 | 13.6 | | ± | 6.0 | | 0.98 | | 0.00 | | |  |  |
| Myostatin [ng∙mL^-1^] | | | | 26.2 | | | ± | 9.4 | | | 30.7 | | | ± | | 7.8 | | | 40.1 | | ± | 4.7 | 28.2 | | ± | 5.9 | | 0.10 | | 0.16 | | |  |  |
| Irisin [ng∙mL^-1^] | | | | 5.5 | | | ± | 2.1 | | | 10.0 | | | ± | | 4.5* | | | 11.7 | | ± | 5.5 | 13.3 | | ± | 10.2 | | **0.00** | | 0.07 | | |  |  |
| Adiponectin [µg∙mL^-1^] | | | | 12.8 | | | ± | 2.5 | | | 17.3 | | | ± | | 6.9 | | | 12.4 | | ± | 1.3 | 19.2 | | ± | 4.9 | | 0.43 | | 0.03 | | |  |  |
| FGF21 [pg∙mL^-1^] | | | | 217.2 | | | ± | 128.2 | | | 239.0 | | | ± | | 297.0 | | | 324.1 | | ± | 170.5 | 184.1 | | ± | 73.7 | | 0.59 | | 0.05 | | |  |  |
| Data are presented as mean± SD; #statistical difference between groups at a time point; *p-*difference between time points; *statistical significance between before and after measurements in the group; $\boldsymbol{\eta}_{\boldsymbol{p}}^{\mathbf{2}}$- effect sizes (partial eta squared): ≥0.01 small, ≥ 0.06 medium and ≥0.14 large effect; HDL-high density lipoprotein; LDL- low density lipoprotein; HOMA- The Homeostasis Model Assessment estimates: HOMA-B- β-cell function; HOMA-S- insulin sensitivity as percentages of a normal reference population and HOMA-IR- insulin resistance | | | | | | | | | | | | | | | | | | | | | | | | | | | | | | | | | | |
| Table S2. Correlation coefficients of irisin and skeletal muscle mass and percent of body fat among YG (n=9) and MG (n=13) group for both acute (after I) and chronic (after X) WBC exposure. | | | | | | | | | | | | | | | | | | | | |  |  |  |  |  |  |  |  |  |  |  |  |  |  |
|  | |  | YG | | | | | | | | MG | | | | | | | | | |  |  |  |  |  |  |  |  |  |  |  |  |  |  |
|  | |  | SMM [kg] | | | | PBF [%] | | | | SMM [kg] | | | | | PBF [%] | | | | |  |  |  |  |  |  |  |  |  |  |  |  |  |  |
|  | |  | before | | | after | before | | | after | before | | | after | | before | | after | | |  |  |  |  |  |  |  |  |  |  |  |  |  |  |
| Irisin  [ng∙mL^-1^] | | before | -0.42 | | |  | 0.20 | | |  | 0.08 | | |  | | 0.58***** | |  | | |  |  |  |  |  |  |  |  |  |  |  |  |  |  |
|  | | 1h after I WBC | 0.47 | | |  | -0.03 | | |  | 0.01 | | |  | | 0.70***** | |  | | |  |  |  |  |  |  |  |  |  |  |  |  |  |  |
|  | | after |  | | | 0.38 |  | | | -0.08 |  | | | 0.09 | |  | | 0.48 | | |  |  |  |  |  |  |  |  |  |  |  |  |  |  |
|  | | 1h after X WBC |  | | | 0.78***** |  | | | 0.05 |  | | | -0.15 | |  | | 0.58***** | | |  |  |  |  |  |  |  |  |  |  |  |  |  |  |
|  | | p | 0.12 | | | 0.28 | 0.69 | | | 0.82 | 0.88 | | | 0.59 | | 0.65 | | 0.76 | | |  |  |  |  |  |  |  |  |  |  |  |  |  |  |
| Values are Spearman correlation; * values were significant at p<0.05; *p-*difference between the correlations; SMM -skeletal muscle mass; PBF- percent of body fat | | | | | | | | | | | | | | | | | | | | |  |  |  |  |  |  |  |  |  |  |  |  |  |  |
